# Survival prediction in sigmoid colon cancer patients with liver metastasis: a prospective cohort study

**DOI:** 10.1093/jncics/pkae080

**Published:** 2024-09-20

**Authors:** Shuai Shao, Dan Tian, Mingyang Li, Shanshan Wu, Dong Zhang

**Affiliations:** Department of Immunology, Beijing Clinical Research Institute, Beijing Friendship Hospital, Capital Medical University, Beijing, China; General Surgery Department, Beijing Friendship Hospital, Capital Medical University, Beijing, China; National Clinical Research Center for Digestive Disease, Beijing, China; Department of Immunology, Beijing Clinical Research Institute, Beijing Friendship Hospital, Capital Medical University, Beijing, China; Department of Immunology, Beijing Clinical Research Institute, Beijing Friendship Hospital, Capital Medical University, Beijing, China; National Clinical Research Center for Digestive Disease, Beijing, China; Department of Gastroenterology, Beijing Friendship Hospital, Capital Medical University, Beijing, China; Department of Immunology, Medical Research Center, Beijing Institute of Respiratory Medicine and Beijing Chao-Yang Hospital, Capital Medical University, Beijing, China; Department of Gastroenterology, Beijing Chao-Yang Hospital, Capital Medical University, Beijing, China

## Abstract

**Background:**

Sigmoid colon cancer is a common type of colorectal cancer, frequently leading to liver metastasis. Predicting cause-specific survival and overall survival in patients with sigmoid colon cancer metastasis to liver is challenging because of the lack of suitable models.

**Methods:**

Patients with sigmoid colon cancer metastasis to liver (2010-2017) in the Surveillance, Epidemiology, and End Results (SEER) Program were recruited. Patients were split into training and validation groups (7:3). Prognostic factors were identified using competing risk and Cox proportional hazards models, and nomograms for cause-specific survival and overall survival were developed. Model performance was evaluated with the concordance index and calibration curves, with a 2-sided *P* value less than* *.05 considered statistically significant.

**Results:**

A total of 4981 sigmoid colon cancer with liver metastasis patients were included, with a median follow-up of 20 months (interquartile range [IQR] = 9-33 months). During follow-up, 72.25% of patients died (68.44% from sigmoid colon cancer, 3.81% from other causes). Age, race, grade, T stage, N stage, surgery, chemotherapy, carcinoembryonic antigen, tumor deposits, lung metastasis, and tumor size were prognostic factors for cause-specific survival and overall survival. The models demonstrated good discrimination and calibration performance, with C index values of 0.79 (95% confidence interval [CI] = 0.78 to 0.80) for cause-specific survival and 0.74 (95% CI = 0.73 to 0.75) for overall survival. A web-based application for real-time cause-specific survival predictions was created, accessible at https://shuaishao.shinyapps.io/SCCLM/.

**Conclusion:**

Prognostic factors for sigmoid colon cancer with liver metastasis patients were identified based on the SEER database, and nomograms for cause-specific survival and overall survival showed good performance. A web-based application was developed to predict sigmoid colon cancer with liver metastasis–specific survival, aiding in survival risk stratification.

Colorectal cancer (CRC) ranks third among the most prevalent malignancies worldwide and the second leading cause of cancer-related deaths (1). Tumor characteristics of CRC differ depending on the tumor location (2). Notably, patients with sigmoid colon cancer have demonstrated the most unfavorable survival outcomes compared with other subsites, accounting for 39.2% of CRC patients (3,4). Liver metastasis is highly prevalent, with approximately 30%-60% of CRC patients developing liver metastasis, indicating the liver as the primary site for CRC metastasis (5). In previous studies, various prognostic factors for CRC patients with liver metastasis, including the American Joint Committee on Cancer stage and metastatic status, were determined through Cox analyses (6). Compared with other subsites, sigmoid colon cancer is more prone to distant metastasis, particularly liver metastasis (7). Despite advancements in multidisciplinary treatment, sigmoid colon cancer patients with liver metastasis mortality rates remain high (8,9). The potential prognostic factors for sigmoid colon cancer with liver metastasis patients are currently unclear. Furthermore, because of individual variations, patients respond differently to treatment, leading to differences in survival rates. Hence, it is necessary to establish an accurate risk prediction model to improve the prognosis for sigmoid colon cancer with liver metastasis patients.

Additionally, there is a lack of large cohort-based studies investigating prognostic factors specifically for sigmoid colon cancer patients with liver metastasis. It is necessary to consider cancer and noncancer factors separately when estimating mortality risk, suggesting the importance of a competing risk model (10). The competing risk model can estimate individual prognostic factors for cancer-specific survival based on patient characteristics, reducing bias, and increasing the accuracy of the results (11,12). To the best of our knowledge, no previous model for cause-specific survival of sigmoid colon cancer with liver metastasis has been constructed yet.

Therefore, we aimed to identify potential prognostic factors and subsequently construct risk prediction models for cause-specific survival as well as overall survival in sigmoid colon cancer with liver metastasis patients based on the Surveillance, Epidemiology, and End Results (SEER) database.

## Methods

### Study population

This study complies with Transparent Reporting of a Multivariable Prediction Model for Individual Prognosis or Diagnosis guidance on multivariable prediction models (13). In this population-based cohort study, we used SEER*Stat software, version 8.4.2, to access the SEER 18 registries, including data for approximately 26.5% of the US population (based on 2020 census). The data underlying this article are available in SEER, and the datasets were derived from sources in the public domain (https://seer.cancer.gov/). We obtained ethical approval for this study from the ethical committee of the Beijing Friendship Hospital (2024-P2-099). Written informed consent was waived, considering the utilization of data from a public database.

**Figure 1.**
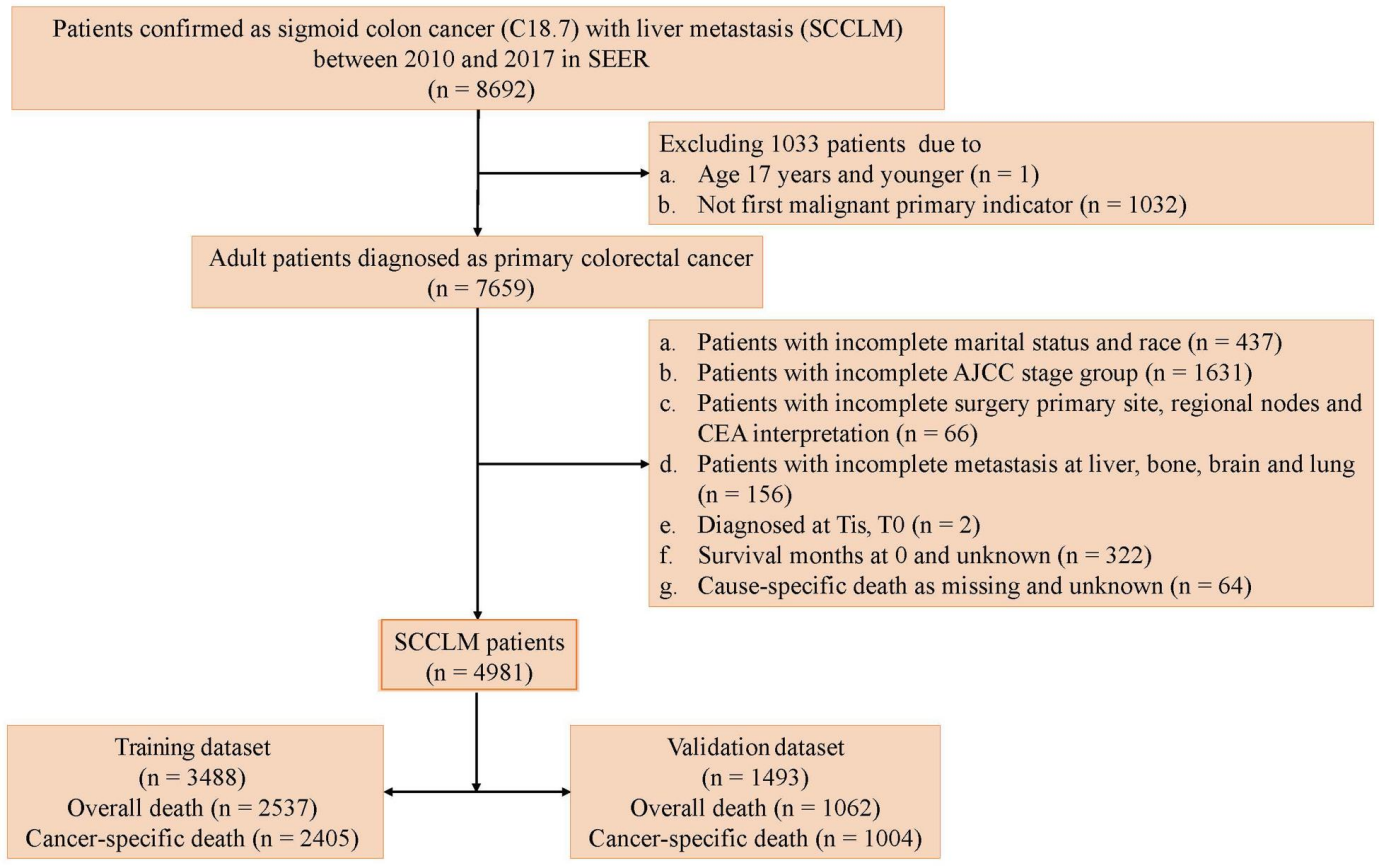
Flow chart for study population. AJCC = American Joint Committee on Cancer; CEA = carcinoembryonic antigen; SCCLM = sigmoid colon cancer with liver metastasis; SEER = Surveillance, Epidemiology, and End Results; Tis = carcinoma in situ.

Patients first primarily diagnosed with sigmoid colon cancer (*International Classification of Diseases* code: C18.7) between 2010 and 2017 in the SEER database were included in the study. The specific inclusion criteria were as follows: 1) aged 18 years and older; 2) pathological or imaging diagnosis of liver metastasis; 3) sigmoid colon cancer as a single primary tumor; and 4) completed information on the survival time and status. The exclusion criteria were as follows: 1) aged younger than 18 years; and 2) incomplete clinical or pathological data, such as age, race, grade of differentiation, T stage, and surgical status. Overall, the 4981 patients were randomly divided into a training group (n = 3488) and a validation group (n = 1493) via 7:3 ratio. The flowchart of participants selection is shown in Figure 1.

### Outcome ascertainment

Outcome of interests were overall death and cancer-specific death. The causes of death were coded according to *International Classification of Diseases Tenth Revision*, which were classified into cancer-specific death and other-cause death. The latter included diseases of heart, cerebrovascular diseases, and other specific causes (Supplementary Figure 1, available online). Overall survival at 1 year, 2 years, and 3 years was defined as the percentage of patients who were still alive, regardless of the cause of death 1, 2, and 3 years after the initial diagnosis. Cause-specific survival at 1 year, 2 years, and 3 years was defined as the percentage of patients who were still alive and had not died from cancer-specific causes 1, 2, and 3 years after the initial diagnosis, respectively.

### Data collection and follow-up

At the baseline, we collected demographic characteristics (age at diagnosis, sex, marital status at diagnosis, race), tumor morphology details (grade of tumor differentiation, primary site, histological type, T stage, N stage, tumor size), clinical variables (carcinoembryonic antigen [CEA] levels, perineural invasion, tumor deposits, lymph node metastasis, metastatic status [metastasis to bone, metastasis to lung, and metastasis to brain]), and therapeutic details (primary tumor surgery, radiotherapy, chemotherapy). In particular, patient marital status was classified as married, single, divorced, and other; race was classified as Black race, White race, and Others (American Indian/AK Native, Asian/Pacific Islander); tumor grade was classified into grade 1 (well differentiated), grade 2 (moderately differentiated), grade 3 (poorly differentiated), and grade 4 (undifferentiated); histologic type was classified as adenocarcinomas, and others; T stage was classified as T1-T2, T3, T4, and undefined; N stage was classified as N0, N1, N2, and undefined; surgery of primary site was defined as not undergone surgery, local tumor excision or partial colectomy or segmental resection (code 10-32), subtotal colectomy or hemicolectomy or total colectomy or proctocolectomy (code 40-70), and surgery (not otherwise specified, NOS); undergone radiation or not; undergone chemotherapy or not; CEA was positive, negative, and undefined; perineural invasion was positive, negative, and undefined; tumor deposit was positive, negative, and undefined; lymph node metastasis was positive negative, and undefined; metastasis to bone or not; metastasis to brain or not; metastasis to lung or not; and tumor size was classified as no more than 4 cm, more than 4 cm, and unclear.

The follow-up period was from the date of diagnosis as sigmoid cancer with liver metastasis until death or censored on December 31, 2017, whichever came first.

### Web-based application

We developed an interactive web-based application using the Shiny package (version 1.8.1.1) in R to enhance the clinical applicability of our prognostic model. The fluid page layout includes a sidebar for input parameters (numeric fields for age, dropdown menus for race, tumor grade, T stage, N stage, etc) and a main panel for displaying results. The application generates a visual nomogram and calculates predicted 1-year, 2-year, and 3-year cancer-specific survival probabilities, also displaying total points from the nomogram.

### Statistical analysis

Quantitative data were summarized by mean and standard deviation, and differences between the training and validation groups were analyzed using *t* test or Mann–Whitney test. Categorical data were characterized as frequency (%) and compared using χ^2^ test. Kaplan–Meier method was conducted to calculate the incidence of overall mortality, and cumulative incidence function was used to calculate cancer-specific mortality. Univariable and multivariable Cox regression were conducted to identify independent prognostic factors for overall survival. The Fine–Gray competing risk model was conducted to identify prognostic factors affecting cause-specific survival. Nomograms were constructed to predict cause-specific survival and overall survival in patients with sigmoid colon cancer metastasis to liver. Model discrimination was evaluated using the Harrell consistency index (C index) with 95% confidence interval. Calibration was evaluated graphically by plotting observed vs predicted probability in deciles of predicted probability.

All statistical analyses were performed by R4.3.0. A 2-sided *P* value less than .05 was considered statistically significant.

## Results

### Baseline characteristics of sigmoid colon cancer with liver metastasis patients

The clinicopathological information for all patients was presented in Table 1. We included 4981 sigmoid colon cancer with liver metastasis patients. The mean age at diagnosis was 60.14 (standard deviation 13.13) years. There was a higher percentage of male patients (58.9%) with predominantly White race. Grade 2 tumors accounted for 63.4% of patients. There were 98.1% of patients diagnosed with adenocarcinoma. Tumors at T3 and T4 stages were predominant, constituting 41.1% and 32.2%, respectively. Regarding lymph node involvement, N0, N1, and N2 stages made up 31.8%, 36.1%, and 27.8%, respectively. Patients with a tumor diameter greater than 4 cm constituted 53.5% of the cases. Regarding treatment, 68.8% of patients underwent surgery, only 5.3% received radiotherapy, and 77% underwent chemotherapy. Among these patients with sigmoid colon cancer liver metastasis, 66.5% tested positive for CEA, 21.8% exhibited neural invasion, and 23.6% had tumor deposits. Among those patients, 51.3% developed lymph node metastasis, while occurrences of bone metastasis, brain metastasis, and lung metastasis were 4.1%, 0.8%, and 20%, respectively.

**Table 1.** Baseline characteristics of the patients with sigmoid colon cancer metastasis to liver

Characteristics	Training set	Testing set	Overall	Train vs test, t/χ^2^	*P*
	(n =* *3488)	(n* *=* *1493)	(n* *=* *4981)		
Age, mean (SD), y	60.08 (13.02)	60.28 (13.38)	60.14 (13.13)	−0.507	.61
Sex, No. (%)				0.905	.34
Male	2069 (59.3)	864 (57.9)	2933 (58.9)		
Female	1419 (40.7)	629 (42.1)	2048 (41.1)		
Marital status, No. (%)				2.973	.43
Married	1983 (56.9)	822 (55.1)	2805 (56.3)		
Single	764 (21.9)	333 (22.3)	1097 (22)		
Divorced	390 (11.2)	166 (11.1)	556 (11.2)		
Others	351 (10.1)	172 (11.5)	523 (10.5)		
Race, No. (%)				4.84	.089
Black	456 (13.1)	168 (11.3)	624 (12.5)		
Other	391 (11.2)	190 (12.7)	581 (11.7)		
White	2641 (75.7)	1135 (76)	3776 (75.8)		
Grade, No. (%)				1.576	.81
1	143 (4.1)	54 (3.6)	197 (4)		
2	2213 (63.4)	943 (63.2)	3156 (63.4)		
3	471 (13.5)	195 (13.1)	666 (13.4)		
4	86 (2.5)	39 (2.6)	125 (2.5)		
Undefined	575 (16.5)	262 (17.5)	837 (16.8)		
Histology, No. (%)				0.216	.64
Adenocarcinoma	3418 (98)	1466 (98.2)	4884 (98.1)		
Others	70 (2)	27 (1.8)	97 (1.9)		
T stage, No. (%)				2.328	.51
T1-T2	397 (11.4)	185 (12.4)	582 (11.7)		
T3	1445 (41.4)	600 (40.2)	2045 (41.1)		
T4	1132 (32.5)	472 (31.6)	1604 (32.2)		
Undefined	514 (14.7)	236 (15.8)	750 (15.1)		
N stage, No. (%)				5.795	.12
N0	1076 (30.8)	508 (34)	1584 (31.8)		
N1	1288 (36.9)	510 (34.2)	1798 (36.1)		
N2	975 (28)	408 (27.3)	1383 (27.8)		
Undefined	149 (4.3)	67 (4.5)	216 (4.3)		
Surgery,^a^ No. (%)				1.68	.64
None	1070 (30.7)	482 (32.3)	1552 (31.2)		
Local tumor excision or partial colectomy or segmental resection	1890 (54.2)	800 (53.6)	2690 (54)		
Subtotal colectomy or hemicolectomy or total colectomy or proctocolectomy	507 (14.5)	202 (13.5)	709 (14.2)		
Surgery, not otherwise specified	21 (0.6)	9 (0.6)	30 (0.6)		
Radiation, No. (%)				1.608	.21
None	3313 (95)	1405 (94.1)	4718 (94.7)		
Yes	175 (5)	88 (5.9)	263 (5.3)		
Chemotherapy, No. (%)				1.71	.19
None	821 (23.5)	326 (21.8)	1147 (23)		
Yes	2667 (76.5)	1167 (78.2)	3834 (77)		
Carcinoembryonic antigen, No. (%)				6.333	.042
Negative	300 (8.6)	153 (10.2)	453 (9.1)		
Positive	2310 (66.2)	1004 (67.2)	3314 (66.5)		
Undefined	878 (25.2)	336 (22.5)	1214 (24.4)		
Perineural invasion, No. (%)				2.812	.25
No	1738 (49.8)	722 (48.4)	2460 (49.4)		
Yes	771 (22.1)	317 (21.2)	1088 (21.8)		
Undefined	979 (28.1)	454 (30.4)	1433 (28.8)		
Tumor deposits, No. (%)				3.005	.22
No	1397 (40.1)	602 (40.3)	1999 (40.1)		
Yes	845 (24.2)	330 (22.1)	1175 (23.6)		
Undefined	1246 (35.7)	561 (37.6)	1807 (36.3)		
Lymph node metastasis, No. (%)				4.467	.11
No	544 (15.6)	250 (16.7)	794 (15.9)		
Yes	1824 (52.3)	732 (49)	2556 (51.3)		
Undefined	1120 (32.1)	511 (34.2)	1631 (32.7)		
Metastasis to bone, No. (%)				0.487	.49
No	3351 (96.1)	1428 (95.6)	4779 (95.9)		
Yes	137 (3.9)	65 (4.4)	202 (4.1)		
Metastasis to brain, No. (%)				0.772	.39
No	3459 (99.2)	1484 (99.4)	4943 (99.2)		
Yes	29 (0.8)	9 (0.6)	38 (0.8)		
Metastasis to lung, No. (%)				1.184	.28
No	2806 (80.4)	1181 (79.1)	3987 (80)		
Yes	682 (19.6)	312 (20.9)	994 (20)		
Tumor size, No. (%)				0.011	.99
≤4 cm	877 (25.1)	375 (25.1)	1252 (25.1)		
>4 cm	1868 (53.6)	798 (53.4)	2666 (53.5)		
Unclear	743 (21.3)	320 (21.4)	1063 (21.3)		
Survival time, median (IQR)	20 (9-33)	20 (8-34)	20 (9-33)	−0.228	.82

a Local tumor excision or partial colectomy or segmental resection (code 10-32); subtotal colectomy or hemicolectomy or total colectomy or proctocolectomy (code 40-70). IQR = interquartile range.

Generally, no statistically significant differences were observed between the training dataset and the validation dataset (*P *>* *.05).

### Cumulative overall mortality and cancer-specific mortality

The median follow-up period was 20 months (interquartile range [IQR] = 9-33 months). Overall, 72.25% (3599) of the patients died during the whole period, including 68.44% (3409) from sigmoid colon cancer and 3.81% (190) from causes other than sigmoid colon cancer (Supplementary Figure 1, available online). The cumulative cancer-specific mortality at the 1-, 2-, and 3-year intervals was 29.04%, 48.44%, and 64.46%, respectively. Meanwhile, the overall mortality was 30.2%, 50.44%, and 67.24% at the 1-, 2-, and 3-year intervals, respectively (Supplementary Table 1, available online).

### Prognostic factors for cancer-specific survival and overall survival

#### Cancer-specific survival

**Figure 2.**
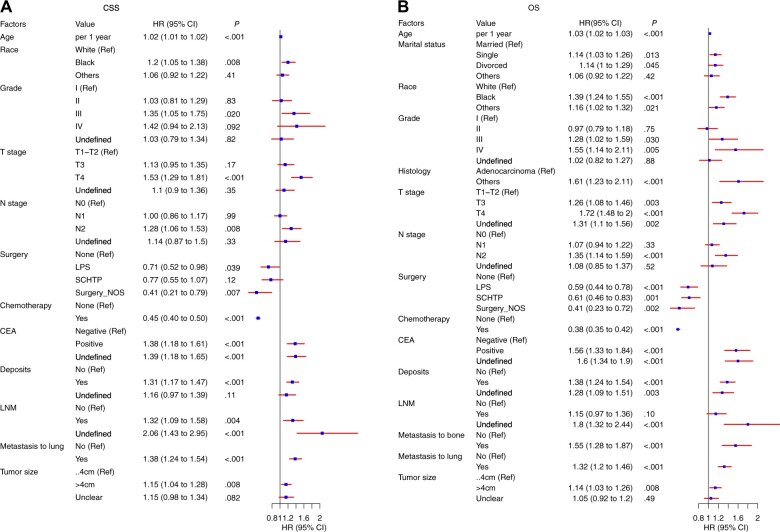
Forest plots illustrating hazard ratios in sigmoid colon cancer patients with liver metastasis, with respect to cancer-specific survival **(A)** and overall survival **(B)**. CI = confidence interval; CEA = carcinoembryonic antigen; CSS = cancer-specific survival; HR = hazard ratio; LNM = lymph node metastasis; LPS = local tumor excision or partial colectomy or segmental resection; NOS = not otherwise specified; OS = overall survival; Ref = referent; SCHTP = subtotal colectomy or hemicolectomy or total colectomy or proctocolectomy; ...4 cm = less than or equal to 4 cm.

Univariable and multivariable competing risk analysis showed that age, race, grade, T stage, N stage, surgery, chemotherapy, CEA, tumor deposits, lymph node metastasis, metastasis to lung, and tumor size (all *P *<* *.05) were statistically significant prognostic factors for cause-specific survival in sigmoid colon cancer with liver metastasis patients, with hazard ratios (HRs) ranging from 1.02 to 2.06. Specifically, each additional year of age, Black race, presenting with grade 3 or higher, T3 or T4 stage, N2 stage, without chemotherapy, CEA positive, tumor deposits positive, metastasis to lymph nodes and lung, and bigger than 4 cm in tumor size were associated with increased cancer specific mortality (CSM) risk, with hazard ratios ranging from 1.01 to 2.95 (Figure 2, A).

#### Overall survival

In addition to age, race, grade, T stage, N stage, surgery, chemotherapy, CEA, tumor deposits, metastasis to lung, and tumor size, other prognostic factors such as marital status, histologic type, and metastasis to bone were identified to positively affect the overall mortality for sigmoid colon cancer with liver metastasis patients, with hazard ratios ranging from 1.06 to 1.61 (Figure 2, B).

### Development of the nomogram prediction model for cancer-specific survival and overall survival

**Figure 3.**
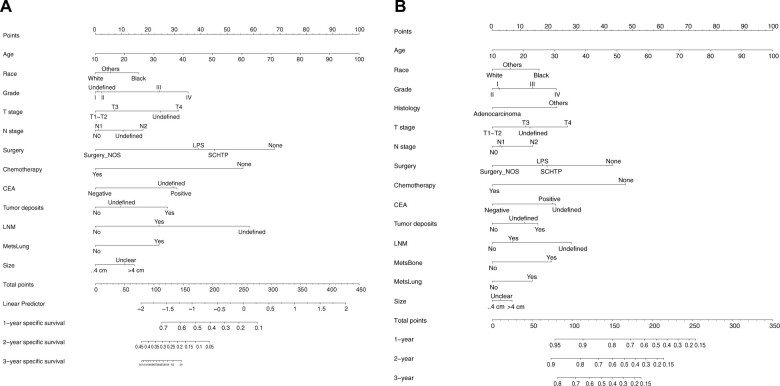
Established nomograms regarding cancer-specific survival **(A)** and overall survival **(B)**. ...4 cm = less than or equal to 4 cm; CEA = carcinoembryonic antigen; LNM = lymph node metastasis; LPS = local tumor excision or partial colectomy or segmental resection; MetsBone = metastasis to bone; MetsLung = metastasis to lung; NOS = not otherwise specified; SCHTP = subtotal colectomy or hemicolectomy or total colectomy or proctocolectomy.

**Figure 4.**
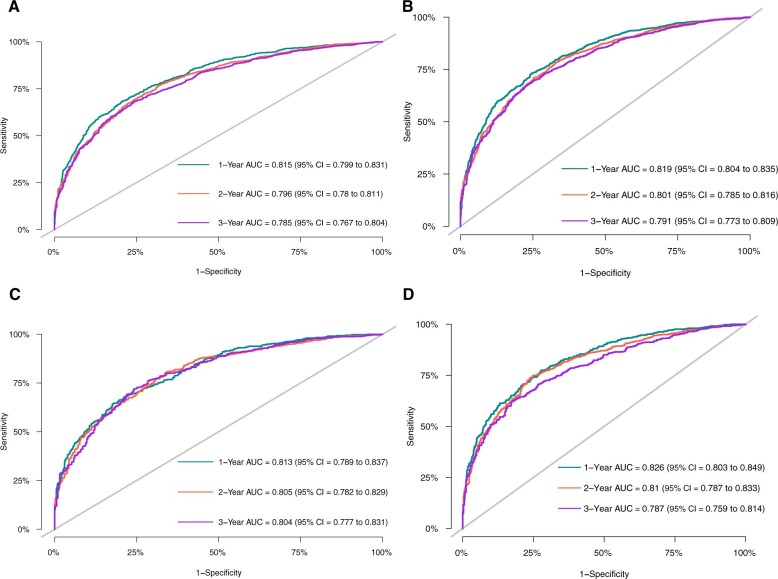
Model discrimination of cancer-specific survival and overall survival in the training dataset and validation dataset in 1 year, 2 years, and 3 years. **A)** Discrimination of cancer-specific survival in the training dataset. **B)** Discrimination of overall survival in the training dataset. **C)** Discrimination of cancer-specific survival in the validation dataset. **D)** Discrimination of overall survival in the validation dataset. AUC = area under the curve; CI = confidence interval.

Nomograms were developed to predict the 1-, 2-, and 3-year cause-specific survival and overall survival for sigmoid colon cancer with liver metastasis patients based on the training dataset (Figure 3, A and B). As for model discrimination, the C index for the cause-specific survival and overall survival during the whole period was 0.790 (95% confidence interval [CI] = 0.784 to 0.796) and 0.741 (95% CI = 0.731 to 0.751), respectively. The area under the receiver operating characteristic curves (AUROCs) for the prediction of cause-specific survival at 1 years, 2 years, and 3 years were 0.815 (95% CI = 0.799 to 0.831), 0.796 (95% CI = 0.78 to 0.811), and 0.785 (95% CI = 0.767 to 0.804), respectively. Similarly, the AUROCs for the prediction of overall survival at 1 year, 2 years, and 3 years were 0.819 (95% CI = 0.804 to 0.835), 0.801 (95% CI = 0.785 to 0.816), and 0.791 (95% CI = 0.773 to 0.809), respectively. In terms of calibration, both models for cause-specific survival and overall survival calibrated well in the training dataset, either in the 1-, 2-, or 3-year prediction (Figure 4, A and B).

### Validation of the cause-specific survival and overall survival prediction models

Validation dataset demonstrated similar model discrimination and calibration for cause-specific survival and overall survival prediction models. Regarding cause-specific survival prediction model, the C index during the whole period was 0.788 (95% CI = 0.779 to 0.796) in the validation group. The AUROCs for the cause-specific survival at 1 year, 2 years, and 3 years were 0.813 (95% CI = 0.789 to 0.837), 0.805 (95% CI = 0.782 to 0.829), and 0.804 (95% CI = 0.777 to 0.831) in the validation group, respectively (Figure 4, C). As for overall survival prediction model, the C index of validation group was 0.741 (95% CI = 0.726 to 0.756). Specifically, the AUROCs for the overall survival at 1 year, 2 years, and 3 years were 0.826 (95% CI = 0.803 to 0.849), 0.81 (95% CI = 0.787 to 0.833), and 0.787 (95% CI = 0.759 to 0.814), respectively (Figure 4, D).

**Figure 5.**
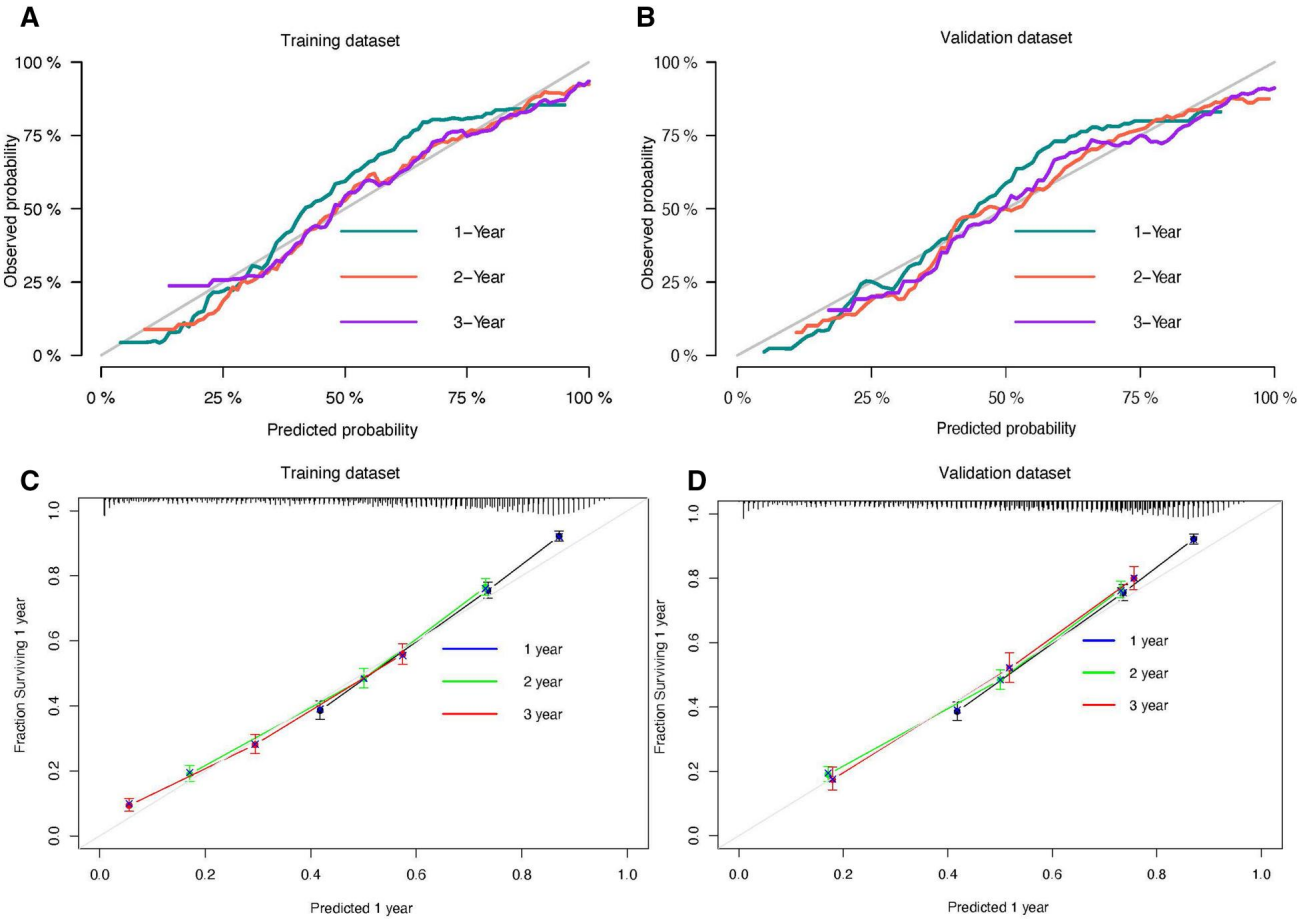
Model calibration of cause-specific survival and overall survival in the training dataset and validation dataset in 1 year, 2 years, and 3 years. **A)** Calibration of cause-specific survival in the training dataset. **B)** Calibration of cause-specific survival in the validation dataset. **C)** Calibration of overall survival in the training dataset. **D)** Calibration of overall survival in the validation dataset.

In addition, calibration curves of cause-specific survival and overall survival prediction models demonstrated a close alignment between predicted probabilities and the actual observed probabilities, indicating that both models achieved well performance of calibration (Figure 5).

### Web-based application for prediction of sigmoid colon cancer with liver metastasis survival

The Shiny application successfully translated our statistical model into an interactive, user-friendly interface. The application is now publicly available at https://shuaishao.shinyapps.io/SCCLM/, allowing for broader access and potential external validation.

## Discussion

Sigmoid colon cancer exhibited a high incidence in all colorectal sites, accounting for 20.18% of CRC patients (Supplementary Figure 2, available online). A rate of 10.22% CRC patients were diagnosed with liver, with the sigmoid colon as the most prevalent primary site (Supplementary Figure 3, available online). In this study, we established risk prediction models for cause-specific survival and overall survival in sigmoid colon cancer with liver metastasis patients based on the SEER database, with good discrimination and calibration performance, providing valuable references for the precise early identification of high-risk individuals prone to mortality. As a result, age, race, differentiation grade, T stage, N stage, surgery, chemotherapy, CEA, tumor deposits, lung metastasis, and tumor size were prognostic factors in both models of cause-specific survival and overall survival.

By comparing the competing risk model and Cox analyses in this study, we identified several novel findings. For the treatment, surgery was identified as a protective factor, and surgical procedures were classified into 2 subgroups. A smaller surgical procedure (local tumor excision or partial colectomy or segmental resection) was found to benefit patients by extending overall survival, with a hazard ratio of 0.59 (95% CI = 0.44 to 0.78; *P *<* *.001). In contrast, the hazard ratio for cause-specific survival with local tumor excision or partial colectomy or segmental resection was 0.71 (95% CI = 0.52 to 0.98; *P *=* *.039), indicating a statistically significant benefit, yet its magnitude is less pronounced than for overall survival. Conversely, patients undergoing a larger surgical scope (subtotal colectomy or hemicolectomy or total colectomy or proctocolectomy) had a hazard ratio of 0.77 (95% CI = 0.55 to 1.07; *P *=* *.120) for cause-specific survival, which did not reach statistical significance. The hazard ratio for overall survival in this group was 0.61 (95% CI = 0.46 to 0.83; *P *=* *.001). This may be attributed to the larger surgical scope being associated with greater tissue damage, potentially leading to a poorer prognosis, which is consistent with findings from a study on transverse colon cancer (14). The Cox regression analysis underestimated the risk of subtotal colectomy or hemicolectomy or total colectomy or proctocolectomy because of the presence of competing events; therefore, the results from the competing risk model are more accurate. In addition, intriguing emerging evidence suggests that CEA has been an important tumor marker for the diagnosis and monitoring of recurrence and metastasis (15). In this study, CEA positive was associated with a 56% and 38% increased risk of overall mortality and cancer-specific mortality, respectively, as demonstrated in previous studies (16,17). Another site-specific variable, tumor deposits, is an irregular cancer nodule formed in the adipose tissue surrounding the colon or rectum (4). They are located within the lymphatic drainage of the primary cancer, yet they do not appear in lymph node tissue and lack vascular structure (18). We found patients with tumor deposits exhibited unfavorable prognoses, consistent with findings from previous studies (19).

When developing a predictive model specifically for sigmoid colon cancer with liver metastasis patients, we identified different prognostic factors compared with previous studies on colon cancer patients with liver metastasis. For instance, race, CEA positivity, lymph node metastasis, and tumor size were not considered prognostic factors in previous models for colon cancer patients with liver metastasis, indicating that these 4 factors may serve as specific prognostic indicators for sigmoid colon cancer with liver metastasis patients (20). Because these prognostic factors could be obtained from routine clinical practice, our model may offer higher cost-effectiveness and wider applicability. Furthermore, by utilizing competing risk model, we were able to eliminate false-positive results from the Cox model. We also obtained higher C index and AUROC values compared with previous studies on colon cancer patients with liver metastasis models (21). This enabled us to provide clinicians with more accurate guidance.

To the best of our knowledge, there are no studies reporting the competing-risk model of prognostic factors for sigmoid colon cancer with liver metastasis, specifically using the largest number of sample size and various variables. Additionally, discrimination and calibration were conducted to verify the robustness of the models. Moreover, although patients with sigmoid colon cancer with liver metastasis who underwent surgical treatment experienced a reduced risk of mortality, a larger surgical scope did not confer a statistically significant protective effect. A definitive monitoring of mortality using prediction models will provide valuable insights for clinicians in improving prognosis and taking into account optimal treatment strategies for patients with sigmoid colon cancer with liver metastasis.

A Shiny application was developed to predict the sigmoid colon cancer with liver metastasis–specific survival, enhancing clinical practice by aiding in follow-up planning with readily available prognostic information (22). Despite its potential benefits, it is based on a specific dataset, which may limit generalizability and will require regular updates to maintain accuracy as treatment paradigms evolve, emphasizing the need for its use alongside clinical judgment.

Although the SEER database offered abundant clinical data, limitations existed in our research. Firstly, our study included patients diagnosed between 2010 and 2017, which may introduce potential bias because of the relatively short observation period. Additionally, the stringent exclusion criteria applied in this research were intended to enhance the homogeneity and reliability of the dataset; however, this approach might have inadvertently excluded patients that could have provided valuable insights into the research question. Secondly, there is a paucity of genetic profiles and general information on biomarkers in the SEER database. Thus, we could not explore the prognostic value of these genetic and biomarker profiles. Further thorough assessment and integration of these profiles into the risk prediction model may be helpful to improve the accuracy of risk prediction. Finally, despite the good performance of discrimination and calibration of our constructed models, our results should be interpreted with caution because of the predominantly White ethnicity. Further various external validations are warranted to confirm our findings.

In summary, we established cause-specific survival and overall survival risk prediction models for sigmoid colon cancer with liver metastasis patients based on the SEER database, with good discrimination and calibration performance. Prognostic factors for cause-specific survival and overall survival included age, race, differentiation grade, T stage, N stage, surgery, chemotherapy, CEA, tumor deposits, lung metastasis, and tumor size. A Shiny application based on the cause-specific survival nomogram was developed to predict sigmoid colon cancer with liver metastasis–specific survival (https://shuaishao.shinyapps.io/SCCLM/). Future guidelines may incorporate these findings into clinical practice for optimal individualized follow-up strategies for patients. Prospective cohort studies in diverse populations are warranted for further validation.

## Supplementary Material

pkae080_Supplementary_Data

## Data Availability

The research data are available in the article itself and its Supplementary Material (available online). Other relevant clinical data will be shared upon any request.
