# Supplementary material for: Survival prediction in sigmoid colon cancer patients with liver metastasis: a prospective cohort study
Source: JNCI Cancer Spectr. 2024 Sep 20;8(5):pkae080. doi: 10.1093/jncics/pkae080 (PMC11476935; doi:10.1093/jncics/pkae080)
Supplement: pkae080_Supplementary_Data [file pkae080_supplementary_data.pdf]

Supplementary Materials

Supplementary Table 1 Comparison between competing risk analysis and Cox regression analysis in estimating mortality rates

		Competing risk analysis	
Survival time (year)	Overall death (%)	Cancer-specific death (%)	Death of other causes (%)
1	30.2	29.04	1.96
2	50.44	48.44	2.69
3	67.24	64.46	3.44
4	77.34	74.03	3.87
5	82.72	79.1	4.32

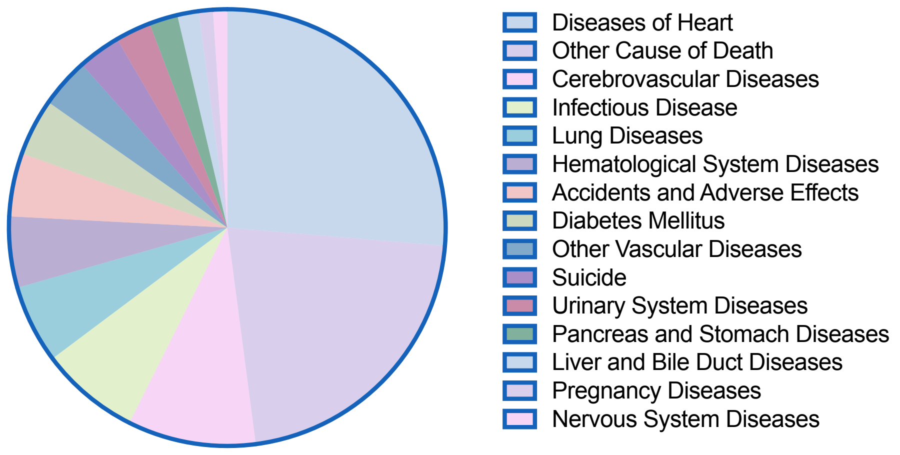

Supplementary Figure 1 The proportion of death of other causes.

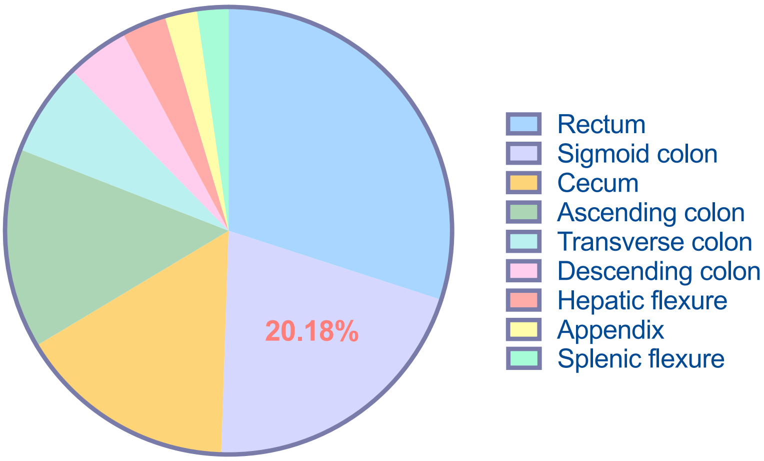

Supplementary Figure 2 The proportion of cancer in various colorectal sites.

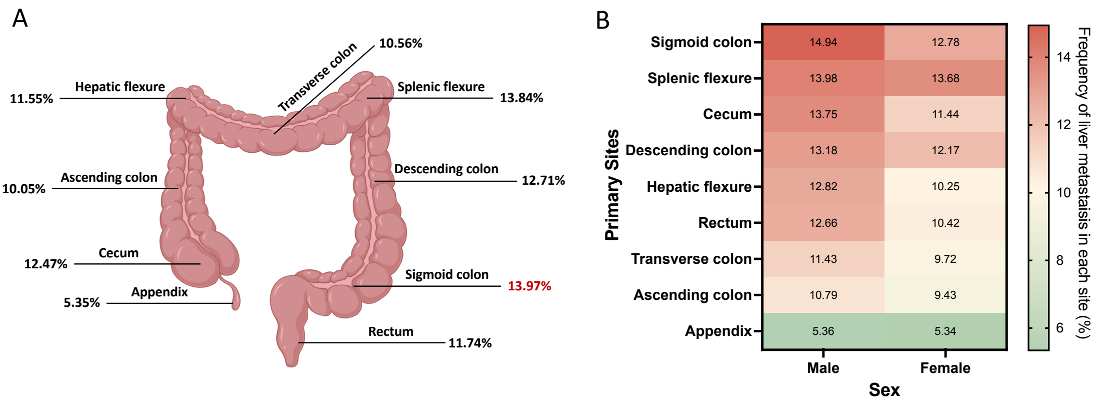

Supplementary Figure 3 Epidemiology of sigmoid colon cancer with liver metastasis. (A) The proportion of liver metastasis in various colorectal segments. (B) The proportion of primary colorectal sites with liver metastasis in both sexes.
